# Association between youth athletes’ sports specialization and injuries: a systematic review and meta-analysis

**DOI:** 10.7717/peerj.21680

**Published:** 2026-09-07

**Authors:** Guannan Lai, Bin-Bin Fang, Weijie Wang, Frank J.H. Lu, Wei-Jiun Shen

**Affiliations:** 1 School of Physical Education, Putian University, Putian, China; 2 School of Physical Education, Quanzhou Normal University, Quanzhou, China; 3 Department of Physical Education, Chinese Culture University, Taipei, Taiwan; 4 Graduate Institute of Physical Education, National Taiwan Sport University, Taoyuan, Taiwan

**Keywords:** Competitive sports, Youth sport specialization, Sports injury, Adolescent athletes, Injury heterogeneity

## Abstract

**Introduction:**

The purpose of this study was to conduct a systematic review and meta-analysis to examine the specialization–injury relationship, and explore whether the specialization–injury relationship is moderated by study design, sport type, age, sex, and injury measurement type, injury mechanism, and anatomical location.

**Methods:**

We searched eight databases by related keywords and assessed the quality of the included studies using the JBI Critical Appraisal Checklist for Analytical Cross-Sectional Studies and the Newcastle–Ottawa Scale. The Comprehensive Meta-Analysis (CMA) statistical software 3.7 examined heterogeneity, sensitivity, publication bias, overall effect size of specialization–injury relationship, and moderation effects. This review was prospectively registered in PROSPERO (CRD420251233318). Searches were conducted in eight electronic databases from inception to March, 2026.

**Results:**

The 15 included studies showed moderate heterogeneity, stable sensitivity analyses, and no publication bias. The overall odds ratio of the sports specialization–injury relationship was 2.00 (95% confidence interval [1.58–2.55], *p* < .001). Moderation analyses indicated that sport type significantly influenced the specialization–injury relationship, whereas no significant moderation effects were observed for study design, sex, age, injury measurement type, injury mechanism, and injury anatomical location.

**Conclusions:**

The findings indicate a positive association between sport specialization and injury risk among youth athletes, with variation across sport participation contexts. Specifically, athletes in both contact and non-contact sports demonstrated higher pooled odds of injury than those involved in multiple sports. Although the evidence is heterogeneous and should be interpreted with caution, these findings highlight the potential role of diversified sport participation in relation to injury.

## Introduction

Engagement in competitive sports has been associated with physical, psychological, and social benefits among adolescents (Gill, Williams & Reifsteck, 2017). However, a subset of youth athletes increasingly concentrate on a single sport from an early age, often engaging in year-round, intensive training to pursue high-level performance. Jayanthi et al. (2015) termed this pattern of participation “sport specialization”. Multiple factors contribute to early specialization, including parental expectations, pursuit of scholarships, aspirations for professional careers, and perceived long-term economic advantages in competitive sport contexts (Malina, 2010).

Prolonged and intensive training schedules are commonly adopted to enhance athletic performance; however, such training loads may contribute to adverse physical, psychological, and social outcomes. Reported consequences include overdependence (Cheatham & Little, 2015), burnout (Brenner et al., 2019), social isolation (Popkin, Bayomy & Ahmad, 2019), and increased injury risk (Biese et al., 2020b; Post et al., 2017). High specialization typically involves repetitive loading of the same musculoskeletal structures, which elevates the likelihood of both acute and chronic injuries (Jayanthi et al., 2019; Kliethermes et al., 2021). In response to these concerns, several professional organizations, including the International Olympic Committee (IOC), the American Academy of Pediatrics (AAP), and the American Medical Society for Sports Medicine (AMSSM), have issued recommendations against early specialization, particularly for pre-pubescent athletes younger than 12 years (Bell et al., 2016; Biese et al., 2020a).

Research examining whether sport specialization is associated with injury risk has produced mixed findings across study designs. For example, a large cross-sectional study involving 1,544 high school athletes reported that highly specialized athletes had significantly greater odds of lower extremity injury compared with low-specialization peers (odds ratio (OR) = 2.08; 95% confidence interval (CI) [1.55–2.80]) (Post et al., 2017). Similar patterns have been documented in multiple studies (Bell et al., 2016; Biese et al., 2020a; Biese et al., 2020b; Post et al., 2021; Whatman et al., 2021; Zoellner et al., 2022). In contrast, several studies adopted a case-control design to examine the effects of sport specialization. For example, to identify the incidence of sport-related injuries due to sport specialization, Jayanthi et al. (2015) recruited 822 youth athletes who appeared in medical clinics because of sports injuries. At the same time, they recruited 368 adolescents who visited pediatric clinics for regular medical examinations. Both groups were asked to complete surveys of sport specialization, training, and injury. Compared to non-sport specialization counterparts, sport specialization youth athletes had greater odds of injury (OR = 1.27; 95% CI [1.07–1.52]; *p* < .01). Similar result was also found in other case-control study (*e.g.*, Jayanthi et al., 2020). Given these findings, synthesizing the available evidence to estimate the magnitude of the specialization–injury association is warranted, while recognizing differences in study design and outcome measurement.

In addition, research that examined the specialization–injury relationship recruited youth athletes participating in different sports. For example, Zoellner et al. (2022) recruited 414 New Zealand youth football players (aged 10–15 years) and completed a sports participation questionnaire and history of injury. They found those who trained for more than 8 months of the year had a 2.86 OR of “gradual onset injury” (95% CI [1.17–6.99], *p* < .02). In contrast, Biese et al. (2020a) sampled 1,588 female volleyball players (grades 9th to 12th) who completed a questionnaire package regarding their sports participation and previous history. They found high specialization participants had a higher incidence of injury (OR: 2.30, 95% CI [1.64–3.24]; *p* < .001) than those with moderate specialization (OR: 1.84, 95% CI [1.29–2.62]; *p* < .001) and low specialization. These variations suggest that sport context (*e.g.*, contact and non-contact sports) may influence the observed association, rather than implying a uniform effect across all sports.

Further, studies examining specialization–injury relationships generally recruit participants of different ages and sexes. Whether age and sex can moderate the specialization and injury relationship needs to be examined. According to developmental and pediatric sport science literature, boys typically enter puberty between ages 9 and 14 and girls between 8 and 13, accompanied by substantial physical and biological changes (Biro et al., 2010; Malina et al., 2015). Furthermore, studies that examine specialization–injury relationships may recruit girls, boys, or both. For example, Biese et al. (2020b) sampled 2,920 female volleyball players, while other studies recruit both male and female participants (Bell et al., 2016; Post et al., 2017; Post et al., 2021). Thus, age and sex might moderate the specialization–injury relationship.

Notably, studies investigating the specialization–injury association have utilized two distinct approaches to assess sports injuries: clinical diagnosis and self-report. These methodological differences may introduce variability in outcome capture, potentially moderating observed relationships. Clinical diagnosis, typically performed by healthcare professionals, relies on objective criteria (*e.g.*, physical examinations, medical records) (McGuine et al., 2017), offering precision in identifying acute or severe injuries but potentially undercounting mild, unreported cases. In contrast, self-report measures depend on participant recall *via* surveys (Zoellner et al., 2022), which may overestimate incidence due to subjective interpretation of minor injuries or recall bias. Such discrepancies in measurement rigor and scope could alter the observed link between sport specialization and injury risk. Therefore, examining whether injury measurement type moderates this association is critical to validating the robustness of findings.

Previous meta-analyses that examine the specialization–injury relationship skipped moderation analyses. For example, Bell et al. (2018) conducted a systematic review and meta-analysis. They included five studies and found athletes with high specialization had higher overuse injury relative risk than athletes with low (pooled relative risk ratio = 1.81; 95% CI [1.26–2.60]) and moderate specialization (pooled relative risk ratio = 1.18; 95% CI [1.05–1.33]). Although Bell et al. (2018) framed their analysis in terms of overuse injury, the included primary studies varied in how injury outcomes were defined and operationalized, including self-reported overuse injuries, gradual-onset injuries, and in some cases broader musculoskeletal conditions.

This inconsistency suggests that the construct of “overuse injury” was not uniformly defined across studies, which may limit the interpretability of the pooled estimates. Moreover, recent empirical studies have further expanded the range of injury outcomes examined in relation to sport specialization, including acute injuries, lower extremity injuries, and general sports injuries (Biese et al., 2020b; McGuine et al., 2017; Whatman et al., 2021). Given this variability, prior meta-analyses may not have fully considered the potential influence of heterogeneity in injury definitions and methodological differences (*e.g.*, study design and injury measurement approaches) on the observed specialization–injury relationship.

To further address this issue, the present study exploratively distinguishes injury outcomes according to two complementary classification principles: injury mechanism and anatomical location. First, injury mechanism was classified into overuse injuries and mixed injury outcomes. This distinction is consistent with sport injury surveillance recommendations that classify injuries according to mechanism or mode of onset, including traumatic and overuse-related mechanisms (Fuller et al., 2006; International Olympic Committee Injury and Illness Epidemiology Consensus Group et al., 2020). Overuse injuries generally refer to injuries resulting from repetitive submaximal loading of the musculoskeletal system without sufficient recovery, whereas mixed injury outcomes refer to broader or non-specific categories that cannot be confidently classified as purely overuse-related, including non-specific musculoskeletal injuries, concussions, and stress fractures (DiFiori et al., 2014). Second, injury anatomical location was classified into lower extremity and upper extremity injuries when the injury site was clearly reported. This classification is also consistent with injury surveillance frameworks recommending that injuries be reported by body region or anatomical location to improve comparability across studies (Fuller et al., 2006; International Olympic Committee Injury and Illness Epidemiology Consensus Group et al., 2020). Accordingly, although the present meta-analysis adopts a broader sports injury framework to estimate the overall injury burden associated with sport specialization, it also examines whether the specialization–injury association differs by injury mechanism and anatomical location. This approach allows the pooled analysis to remain comprehensive while directly addressing the clinical heterogeneity of the included injury outcomes.

In general, the present study expands upon Bell et al. (2018) by incorporating a broader range of injury outcomes beyond overuse injury, thereby better reflecting the heterogeneity of injury definitions in the literature. Furthermore, this study extends prior work by systematically examining potential moderators, including study design, sport type, age, sex, and injury measurement type, injury mechanism, and injury anatomical location, to provide a more nuanced understanding of the specialization–injury relationship.

## Materials & Methods

### Article search strategy

The present systematic review was prospectively registered in the International Prospective Register of Systematic Reviews (PROSPERO; CRD420251233318) and conducted in accordance with the Preferred Reporting Items for Systematic Reviews and Meta-Analyses (PRISMA) guidelines (Page et al., 2021). No separate review protocol beyond the PROSPERO registration was prepared, and no amendments were made during the review process. No publicly accessible review protocol is available beyond the PROSPERO registration. A comprehensive literature search was conducted across eight electronic databases, including Google Scholar (*n* = 3,650), PubMed (*n* = 80), SAGE (*n* = 32), Web of Science (*n* = 126), Taylor & Francis Online (*n* = 3), ScienceDirect (*n* = 17), Ovid (*n* = 15), and Scopus (*n* = 126), from their inception to March 20, 2026. Google Scholar records were screened by relevance until no additional eligible studies were identified. We used a predefined set of keywords combined with Boolean operators (OR and AND): (a) adolescent “OR” youth “OR” teenager “OR” young athletes “OR” adolescent athletes “OR” youth sports; (b) sport specialization “OR” athletic specialization “OR” sports expertise “OR” specialization “OR” expertise “OR” specialized training “OR” sport specialization; and (c) sports injuries “OR” athletic injuries “OR” sports trauma “OR” injury “OR” injuries “OR” trauma “OR” athletic injuries. A detailed description of the full search strategy, including database-specific search strings, is provided in the Supplementary File S3. Only peer-reviewed articles published in English were included. Title/abstract screening and full-text eligibility assessment were conducted independently by two reviewers (GNL and BBF). During the full-text screening stage, several studies were excluded due to inconsistencies in the operationalization of sport specialization and outcome measurement. Specifically, some studies defined sport specialization using alternative criteria that were not aligned with the three-item scale proposed by Jayanthi et al. (2015), while others reported injury outcomes using metrics that were not comparable to effect size estimates required for meta-analysis. Studies that reported only prevalence rates or incidence rate ratios without convertible effect sizes were excluded from quantitative synthesis to ensure methodological consistency.

### Eligibility criteria

Sport specialization level was measured based on the three core questions proposed by Jayanthi et al. (2015) (*e.g.*, “quit other sports for the primary sport”, “train >8 months/year in the primary sport”, “regard the primary sport as more important”); some studies adapted or extended this scale according to research scenarios (*e.g.*, Zoellner et al. (2022) used a modified four-item yes/no scale validated in New Zealand youth; Okoruwa et al. (2022) added a six-point extended scale), and specialization was consistently categorized into three levels: high, moderate, and low. To avoid selection bias, the same two investigators (GNL and BBF) formulated and applied further inclusion criteria for the following elements of PECOS: (a) Populations: a population sample of adolescents aged 10–19, in line with the definition of adolescence put forth by the World Health Organization (WHO); (b) Exposure: exposure factors are related to sport specialization, such as level of sport specialization, training time, volume, and intensity, etc.; (c) Comparative groups: included studies should include high specialization, moderate and low specialization athletes; (d) Outcomes: the primary outcome of interest was sports-related injuries, including overuse injuries, acute injuries, and general musculoskeletal injuries. Because the included studies used heterogeneous injury definitions, these outcomes were pooled under a broader sports injury construct to estimate the overall injury burden associated with sport specialization. This pooled estimate was not intended to represent any single injury subtype. To further address this heterogeneity, injury mechanism and anatomical location were coded as additional moderators when sufficient information was available. Data were extracted as reported effect estimates. (e) Study design: includes cross-sectional study, case-control study, and cohort study.

### Data extraction

Two reviewers (GNL and BBF) independently extracted information from each included study. Extracted variables included the first author, publication year, and country; methodological characteristics such as study design and sampling method; participant demographics including sample size, percentage of female participants, and mean age with standard deviation; as well as sport type and injury measurement type, mechanism and anatomical location. Sport specialization, the exposure variable, was categorized using a three-level scale (low, moderate, high) based on the instrument developed by Jayanthi et al. (2015).

Coding of moderators was conducted based on predefined criteria to ensure consistency and reproducibility. Study design was categorized as cross-sectional, case-control, or cohort. Sport type was classified into three categories: contact sports (*e.g.*, soccer, basketball), non-contact sports (*e.g.*, softball, volleyball), and multiple sports, where participants were drawn from more than one sport type, which may include both contact and non-contact sports. The classification of contact and non-contact sports was based on the level of physical contact inherent to the sport, as commonly used in sport injury and epidemiological research (Caine, Di Fiori & Maffulli, 2006). The category of multiple sports was applied to studies in which participants were recruited across different sport types and could not be meaningfully classified into a single sport category. Age was categorized into early adolescence (10–13 years) and middle-to-late adolescence (14–18 years), reflecting commonly used developmental stages in adolescent research (Sawyer et al., 2012). When studies included a wide age range spanning both categories, classification was based on the reported mean age of the sample. Sex was coded as male or female based on the reported sample characteristics. Injury measurement type was classified into three categories: clinical diagnosis, self-report, and mix. Clinical diagnosis referred to injuries assessed through medical evaluation or objective diagnostic evidence (*e.g.*, physician diagnosis or medical records), whereas self-report referred to injuries reported directly by participants. The mix category included studies that incorporated both approaches, either by combining clinical and self-reported data or by using cross-validation between self-report and objective diagnostic sources. This variable was incorporated into the moderator analysis as a three-level categorical moderator, and studies were grouped according to their injury ascertainment method to examine whether the association between sport specialization and injury risk differed across clinical diagnosis, self-report, and mixed measurement approaches. Injury mechanism was categorized into two subgroups: mixed injury outcomes and overuse injuries. Mixed injury outcomes included non-specific musculoskeletal injuries, concussions, and stress fractures, representing a mix of traumatic and overuse-related injuries. Overuse injuries referred to chronic overload-related, gradual-onset, and serious overuse injuries caused by repetitive microtrauma without an acute traumatic event. Injury anatomical location was classified as lower extremity injuries or upper extremity injuries. All coding decisions were independently verified by two reviewers, and discrepancies were resolved through discussion.

For quantitative synthesis, reported effect sizes were recorded, specifically OR with corresponding 95% CI. When more than one effect estimate was provided, estimates comparing high *versus* low specialization were prioritized to maintain analytical consistency. All reported sports injury outcomes, regardless of follow-up duration, were extracted when available. Discrepancies between the two reviewers were resolved through discussion and, when necessary, consultation with a third reviewer (FJL). During the data extraction process, two studies were identified as having overlapping samples with other studies (*i.e.,* McGuine et al., 2017; Post et al., 2017). To address this issue, only one study from each overlapping dataset was retained for inclusion in the meta-analysis based on relevance and data completeness, while the corresponding overlapping studies were excluded to ensure independence of effect sizes. This approach minimizes the risk of double-counting participants and improves the validity of pooled estimates. The literature screening was conducted between September 2025 and March 2026, and data extraction was completed in March 2026.

### Article quality assessment

Study quality was independently assessed by two reviewers (GNL and BBF) according to the following criterion: (a) The JBI Critical Appraisal Checklist for Analytical Cross-Sectional Studies from the Joanna Briggs Institute (JBI) (Aromataris et al., 2024) was used to assess the methodological quality of the included cross-sectional studies. The JBI critical appraisal tool comprises eight questions evaluating the clarity of participant selection, the validity and reliability of measurements, the appropriateness of statistical analyses, and consideration of confounding factors. Each item is rated as yes, no, unclear, or not applicable. Studies with more than five “yes” ratings were classified as high quality, those with three to five “yes” ratings as moderate quality, and those with fewer than three “yes” ratings as low quality (Moola et al., 2024). Only studies classified as high quality were included in the meta-analysis. (b) The Newcastle–Ottawa Scale (NOS): The Newcastle Ottawa Scale was applied to evaluate the quality of case-control and cohort studies (Wells et al., 2000). The NOS checklist contains eight items organized into three domains: Selection, Comparability, and Exposure or Outcome. Each eligible item receives one asterisk, with the exception of the Comparability domain, which may receive up to two asterisks for case-control studies. Higher total scores indicate higher methodological quality. Only studies receiving six or more asterisks were retained for inclusion in the meta-analysis (Gong et al., 2023). Based on these criteria, a total of fifteen studies were deemed eligible for the final quantitative synthesis (see Table 1).

**Table 1:** Characteristics of studies included in the meta-analysis of sport specialization and sports injuries.

Author (Year), Country	Participants (*n*, % Female), Mean age (Years)	Study design	Sport type	Specialization measurement	Injury outcome	Injury measurement type	Effect size OR (95% CI)	Main findings
Bell et al. (2016), USA	302 HS athletes 59.6% F, 15.58 ±1.16	Cross-sectional	Contact	Three-point scale	LE musculoskeletal injuries (prospective)	Mix	OI knee: OR = 2.93 (1.16–7.36)	Highly specialized athletes were more likely to report a history of overuse knee or hip injuries. Participating in a single sport for more than 8 months per year appeared to be an important factor in the increased injury risk observed in highly specialized athletes.
Biese et al. (2020a), USA	1,588 volleyball athletes 100% F, 15.6 ±1.1	Cross-sectional	Non-Contact	Three-point scale	Musculoskeletal injuries	Mix	High *vs* Low: OR = 2.30 (1.64–3.23)	Specialization associated with injury history and year-round volleyball opportunities among female adolescent athletes.
Biese et al. (2020b), USA	2,011 adolescent athletes 49.2% F, 13.7 ±1.6	Cross-sectional	Multiple	Three-point scale	Overuse and acute injuries	Mix	High *vs* Low: OI: OR = 1.50 (1.11–2.02)	Highly specialized athletes more likely to report acute and overuse injuries. Association stronger in females.
Buser et al. (2025), USA	485 injured athletes 40.2% F, 16 ±2	Cohort	Multiple	Three-point scale	OI, serious OI (≥30 days RTS)	Clinical Diagnosis	High *vs* Low: Serious OI: OR = 3.19 (1.69–6.03)	Female athletes are more likely to sustain overuse injuries with more organized, year-round, training and less free play compared with their male counterparts
Jayanthi et al. (2015), USA	1,190 young athletes 49.3% F, 13.7 ±2.3	Case-control	Multiple	Three-point scale	OI, serious OI (≥1 month rest)	Clinical Diagnosis	High *vs* Low: Serious OI: OR = 2.25 (1.27–3.99)	There is an independent risk of injury and serious overuse injury in young athletes who specialize in a single sport. Growth rate was not related to injury risk.
Jayanthi et al. (2020), USA	579 youth athletes 53.0% F, 14.1 ±2.3	Case-control	Multiple	Three-point scale	All-cause injuries, OI, serious OI	Clinical Diagnosis	High *vs* Low: OI: OR = 1.46 (1.04–2.04)	Our study confirms that over time, young athletes, and in particular young female athletes, were more likely to be injured and sustain an overuse injury if they had a higher degree of sport specialization.
Lear et al. (2024), USA	1,309 softball players 100% F, 15.1 ±1.7	Cross-sectional	Non-Contact	Three-point and six-point scales	UE overuse (throwing arm)	Self-Report	High *vs* Low: OR = 2.33 (0.97–5.6),	Among female youth softball players, sport specialization has conflicting effects on throwing arm injury; some factors increase/decrease the risk.
McGowan, Whatman & Walters (2019), New Zealand	914 children 59.0% F, 12.6 ±0.5	Cross-sectional	Multiple	Adapted four-item scale Low/Mod/High	Gradual onset injuries	Mix	High *vs* Low: Gradual onset: OR = 0.75 (0.49–1.15)	Early specialization did not increase injury odds. Exceeding sport volume recommendations increased gradual onset injury odds.
Okoruwa et al. (2022), Canada	219 female HS athletes 100% F, NR (13–18)	Cross-sectional	Multiple	Three-point and six-point scales	Stress fractures, concussions	Self-Report	High *vs* Low:OR = 2.93 (1.38–6.24)	Higher specialization associated with greater risk of injuries, concussions, and stress fractures in female athletes.
Post et al. (2017), USA	1,544 HS athletes 50.5% F, 16.1 ±1.1	Cross-sectional	Contact	Three-point scale	Lower extremity injuries	Mix	High *vs* Low: OR = 2.58 (1.88–3.54)	Moderate and high specialization significantly increased LEI risk. Female athletes and larger schools had higher specialization rates.
Post et al. (2020a), USA	716 youth athletes 70.8% F, 14.2 ±1.5	Cross-sectional	Multiple	Three-point scale	Overuse injuries	Mix	High *vs* Low: OR = 1.20 (0.75–1.90)	Sport specialization was not associated with overuse injury in basketball or soccer athletes; female basketball athletes were nearly 4 times more likely to report such injury than males, and youth athletes’ sex and sport may influence specialization-related overuse injury risk.
Post, Struminger & Hibberd (2020b), USA	551 baseball athletes 0% F, 15.8 ±1.3	Cross-sectional	Non-Contact	Three-point scale	UE overuse (shoulder/elbow)	Mix	All: OR = 3.77 (1.39–10.20) Pitchers: OR = 4.29 (1.22–15.10)	We found that being highly specialized in baseball was associated with UE overuse injury history and worse throwing-arm health in high school baseball athletes.
Post et al. (2021), USA	805 basketball players 29.9% F, 12.9 ±2.5	Cross-sectional	Contact	Adapted four-item scale Low/Mod/High	Basketball-related musculoskeletal injuries	Self-Report	High *vs* Low: OR = 2.47 (1.25–4.88)	Specialization in basketball, along with several other behaviors that have become typical of modern youth sport par ticipation, were associated with reported injury history.
Whatman et al. (2021), Canada	238 junior HS students 51.0% F, 12.6 ±NR	Cross-sectional	Multiple	Three-point scale	Musculoskeletal injuries	Mix	High *vs* Low: OR = 2.21 (0.43–11.36)	Sport specialization was present among Canadian junior high school students, though less common than previously reported, and was not associated with sex, grade, school size, history of injury, or a range of physical performance measures.
Zoellner et al. (2022), New Zealand	340 football players 49.0% F, 12.8 ±1.1	Cross-sectional	Contact	Adapted four-item scale Low/Mod/High	Gradual onset injuries	Mix	High *vs* Low: Gradual onset: OR = 4.17 (1.48–11.74)	There is a high prevalence of sport specialization in youth football, and it is associated with increased incidence of gradual onset injury.

**Notes.**

SS Sport Specialization UE Upper Extremity OI Overuse Injury AI Acute Injury F Female HS High School LE Lower Extremity LEI Lower Extremity Injury OR Odds Ratio NR Not Reported

Mix: combining clinical diagnosis and self-report; three-point scale: this instrument was a three-level evaluation scale (Low/Moderate/High) based on Jayanthi et al. (2015).

Detailed quality assessment results are provided in Supplementary Table S1.

### Meta-analytic strategies

Studies were included in each synthesis based on the availability of comparable effect estimates and relevant study-level characteristics. All eligible studies contributed to the overall meta-analysis, whereas subgroup and moderation analyses included only studies reporting the corresponding moderator variables (*e.g.*, study design, sport type, age group, sex, or injury measurement type). The Comprehensive Meta-Analysis (CMA) statistical software version 3.7 was used to examine the association between sport specialization and injury across the fifteen included studies. Following the approach adopted in the study by Bell et al. (2018), only OR and their corresponding CI were entered as effect size inputs. All included studies reported odds ratios with corresponding 95% confidence intervals; therefore, no conversion from other effect size metrics (*e.g.*, hazard ratios or risk ratios) was required in the present analysis. If multiple effect estimates were available within a single study, the comparison between high and low specialization levels was selected to ensure consistency across studies. A random effects model was applied to account for between study heterogeneity and to generate pooled effect estimates.

We assessed heterogeneity using the *Q* test and the *I*^2^ statistic. The *Q* test was applied to determine whether statistically significant heterogeneity was present, whereas the *I*^2^ statistic quantified the magnitude of heterogeneity, with values of 25 percent, 50 percent, and 75 percent interpreted as low, moderate, and high heterogeneity, respectively. Publication bias was evaluated using funnel plot inspection (Verhagen & Ferreira, 2014) as well as Egger’s test and Begg’s test. To examine the influence of individual studies on the pooled effect size, a sensitivity analysis was performed using the leave one out method, in which each study was removed sequentially and the analysis repeated to assess the stability of the results. A forest plot was generated to present the ORs and their corresponding 95% CI, thereby illustrating the association between sport specialization and injury risk (Field & Gillett, 2010).

Finally, moderation analyses were conducted to investigate whether study level characteristics accounted for variability in effect sizes. Based on the data structure of the included studies, subgroup analyses were carried out to examine potential moderators of the specialization injury association. The comprehensive audit is provided in Supplementary: Audit table.

## Results

### Study selection

**Figure 1:**
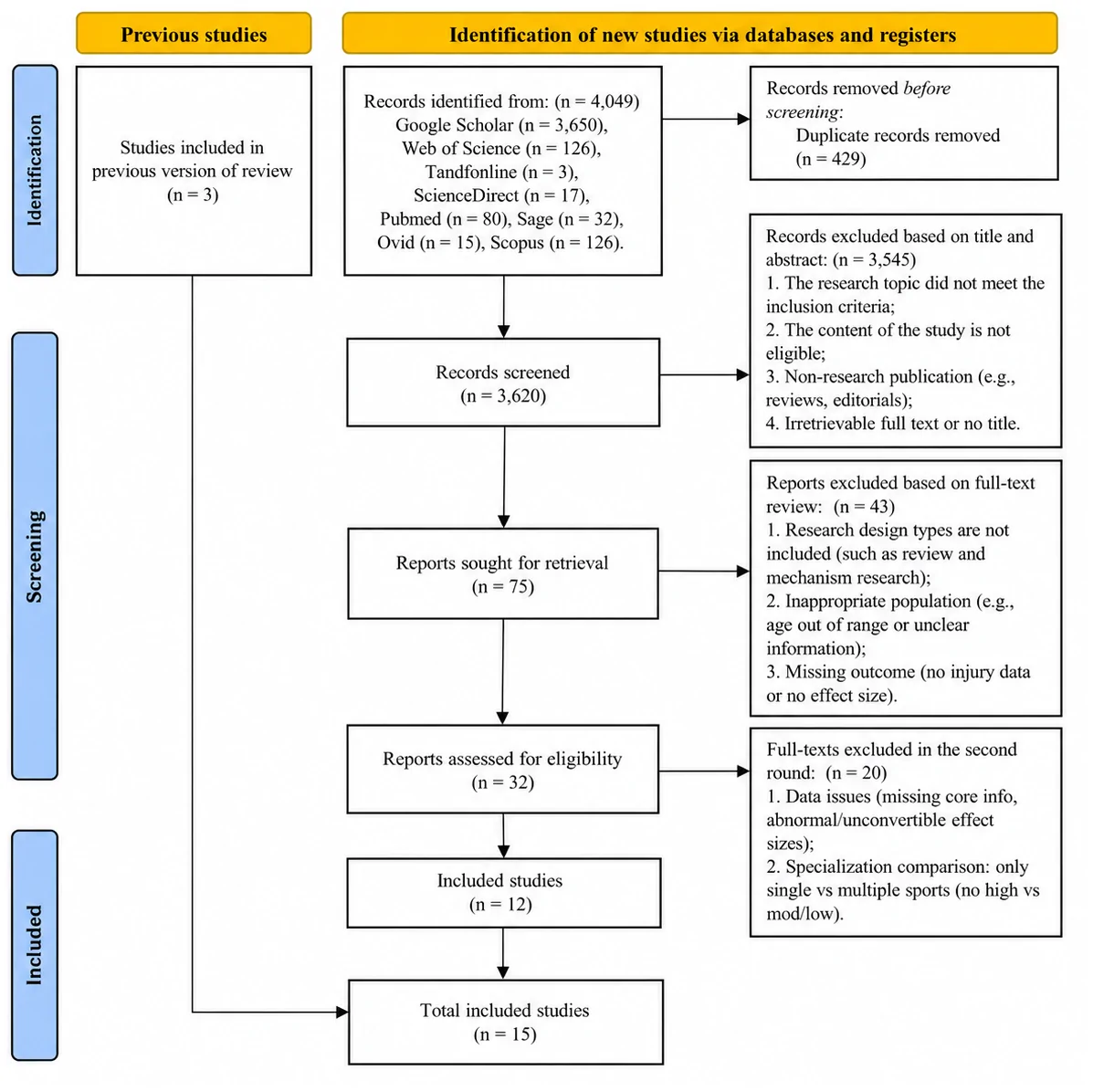
PRISMA flow chart of the studies included in the systematic review and meta-analysis.

Following the initial literature search, a total of 4,049 records were identified from databases and registers. After removing 429 duplicates, 3,620 records were screened based on title and abstract. During this phase, 3,545 records were excluded for the following reasons: the research topic was irrelevant, the study content was ineligible, the publication was a non-research article (*e.g.*, review or editorial), or the full text was irretrievable or the record lacked a title. Subsequently, 75 full-text articles were sought for retrieval and assessed for eligibility. Of these, 43 articles were excluded after full-text review due to: (1) ineligible study designs (*e.g.*, reviews or mechanistic studies), (2) inappropriate population (*e.g.*, age outside the defined range or unclear demographic information), or (3) missing outcomes (*e.g.*, no injury data or unavailable effect sizes). A second round of full-text screening further excluded 20 articles for the following reasons: data issues (*e.g.*, missing core information, abnormal or unconvertible effect sizes), inappropriate specialization comparisons (*e.g.*, only comparing single-sport *vs.* multi-sport participation without distinguishing high *vs.* moderate/low specialization), or duplicate study samples. Ultimately, 12 articles were included in the systematic review. In addition, three studies were incorporated from previous reviews, resulting in a total of 15 studies being included in the final meta-analysis (Fig. 1). All studies that met the predefined inclusion criteria after full-text screening were included in the final synthesis, and no additional eligible studies were excluded at later stages.

### Descriptions of study characteristics

The included 15 studies were published between 2015 and 2025, with 13 released in 2015–2023 and two studies marked with the latest year. Study designs included 12 cross-sectional studies, two case-control studies, and one cohort study: Buser et al. (2025) (6-month injury follow-up). Eric G. Post (USA) was the most prolific author, contributing four studies (Post et al., 2017; Post et al., 2020a; Post, Struminger & Hibberd, 2020b; Post et al., 2021) focused on specialization-related injury risks in youth basketball, baseball, and volleyball. Geographically, 11 studies were from the USA, with two each from New Zealand (McGowan, Whatman & Walters, 2019; Zoellner et al., 2022) and Canada (Whatman et al., 2021; Okoruwa et al., 2022). The final sample included 12,791 athletes (mostly 12–18 years old), with 7,729 females (60.4%) and 5,062 males (39.6%). See Table 1 for details. Overall, the methodological quality of the included studies was rated as moderate to high. All cross-sectional studies met the predefined JBI quality criteria for high quality, and all case-control and cohort studies achieved at least six stars on the Newcastle–Ottawa Scale (see Table S1).

### Heterogeneity analysis

The meta-analysis of the fifteen included studies indicated moderate heterogeneity. The *Q* statistic was 41.11 (*p* < .001), the *I*^2^ value was 65.94%, and the *τ*^2^ value was 0.13. These indicators collectively suggested the presence of moderate heterogeneity. Such heterogeneity may have resulted from methodological and sample differences across studies, including data sources, study design, participant characteristics, study duration, and measurement approaches. Additional variability may also be attributed to factors such as participant age, sex, sport type, and injury characteristics. Consequently, a random effects model was applied, and moderation analyses were conducted to further explore potential sources of heterogeneity.

### Sensitivity analysis

The sensitivity analysis was conducted to assess the robustness of the pooled effect size. Using the leave one out procedure, each study was sequentially removed and the meta-analysis repeated to evaluate the influence of individual studies. The resulting odds ratios ranged from 1.97 to 2.12, compared with the pooled odds ratio of 2.00 (95% CI [1.58–2.55]). These findings indicated that no single study disproportionately affected the overall estimate, demonstrating that the results were stable and robust.

### Publication bias

**Figure 2:**
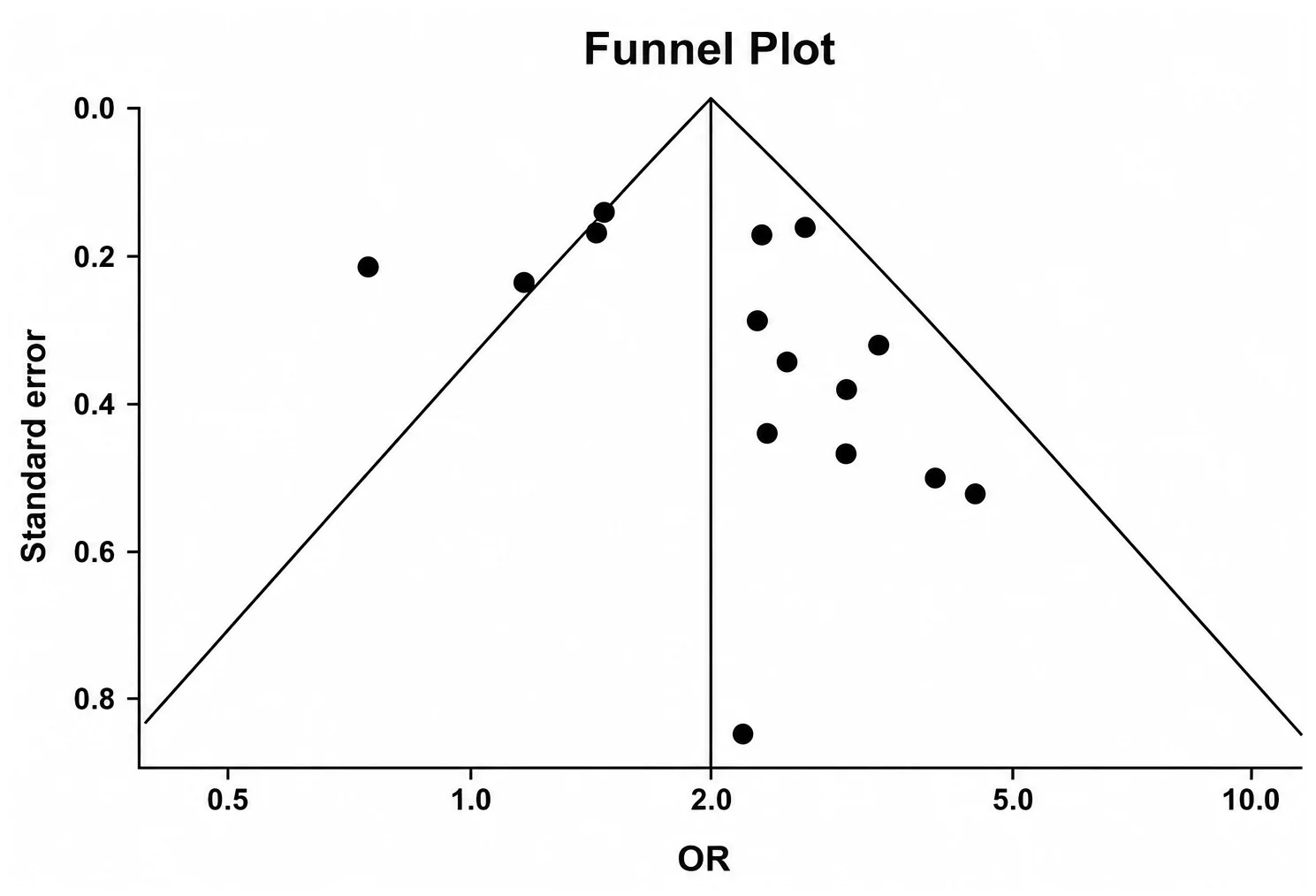
Funnel plot for assessing publication bias in the meta-analysis of sport specialization and injury risk.

Publication bias was first assessed visually using a funnel plot (Fig. 2). Although interpretation of funnel plot symmetry may be limited when the number of included studies is small, the distribution showed no substantial asymmetry around the pooled effect size despite slight imbalance, suggesting no apparent indication of publication bias. Statistical tests were then conducted for further evaluation. Egger’s regression test yielded a beta of 1.47 (standard error = 1.00, *t* (13) = 1.47, *p* = 0.17), indicating no statistically significant evidence of publication bias. Similarly, Begg’s rank correlation test produced a Kendall’s score of 19.00 (standard error = 20.21, *z* = 0.94, *p* = 0.35), further supporting the absence of significant publication bias. Overall, both visual inspection and statistical testing suggested that the pooled estimates were unlikely to be materially affected by publication bias, although these findings should be interpreted with caution given the relatively small number of included studies.

### Main effects of specialization–injury relationship

**Figure 3:**
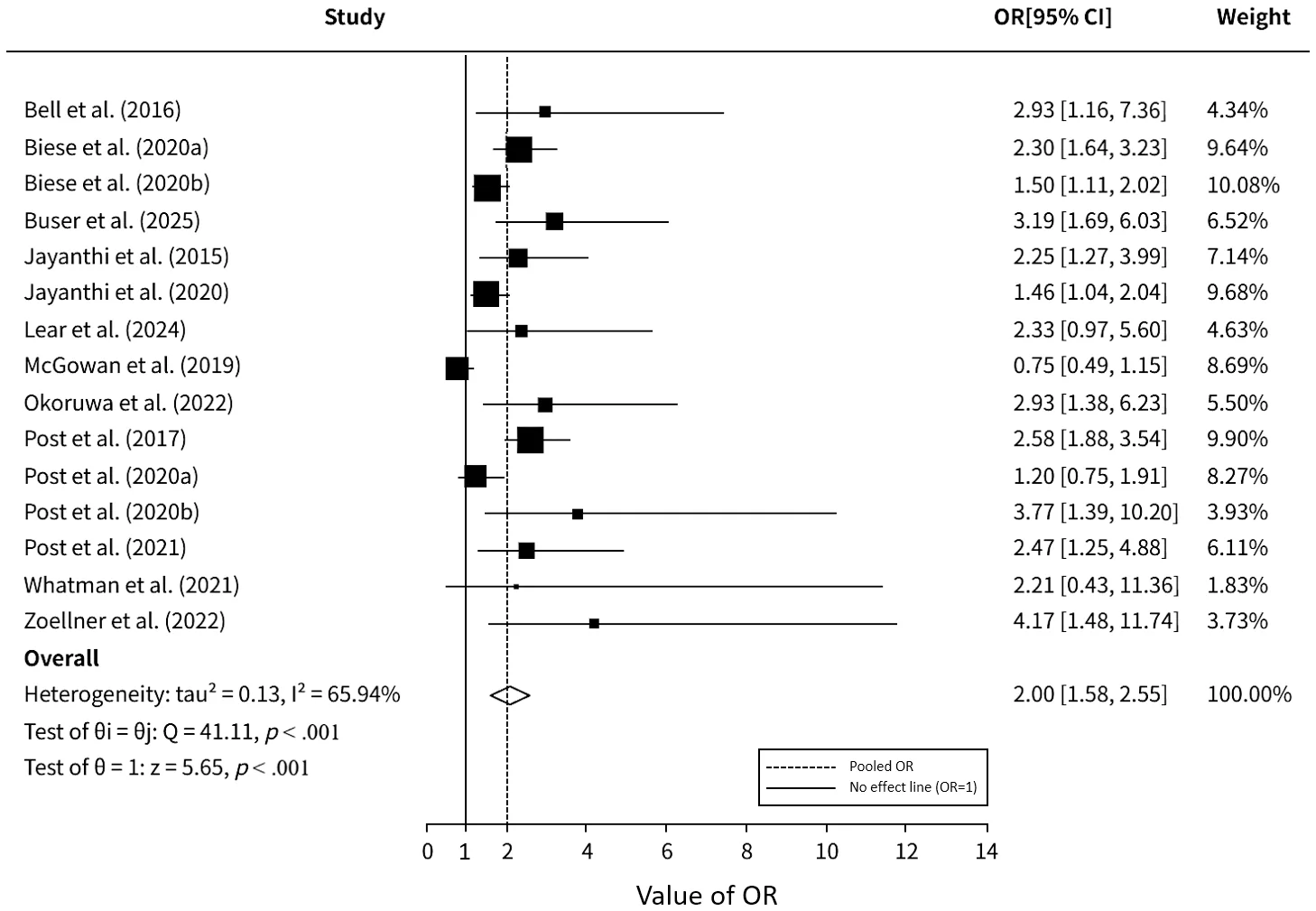
Forest plot of meta-analysis on sport specialization and injury risk.

A summary of the random effects meta-analysis is presented in Fig. 3. Compared with the low level of specialization, higher levels of sport specialization were associated with greater odds of sports injury, and the OR is 2.00 (95% CI [1.58–2.55], *p* < 0.001), indicating that the odds of injury were approximately doubled.

### Moderation analyses

We also examined the moderating effects on the specialization–injury relationship with moderation analyses including the following variables: study design (cross-sectional, cohort, and case-control), sports type (contact sports, non-contact sports, and multiple), age (early adolescence: 10–13; middle-late adolescence: 14–18), sex (female and male), and injury measurement type (clinical diagnosis, self-report and mixed measurement), injury mechanism (overuse injuries and mixed injuries), and injury anatomical location (lower extremity and upper extremity). The corresponding moderation analysis figures are provided in the Supplementary: Figures (Figs. S1–S7).

### The moderating effect of study design

The moderating effect of study design on the specialization–injury relationship was evaluated by categorizing studies into three subgroups: case-control, cohort, and cross-sectional. For the case-control subgroup, the pooled OR was 1.70 (95% CI [1.13–2.55]) with low-to-moderate heterogeneity (*τ*^2^ = 0.04, *I*^2^ = 38.61%). The cohort subgroup included only one study (Buser et al., 2025), with an OR of 3.19 (95% CI [1.69–6.03]). The cross-sectional subgroup had a pooled OR of 2.01 (95% CI [1.49–2.70]) with substantial heterogeneity (*τ*^2^ = 0.16, *I*^2^ = 69.40%). The overall pooled OR across all study designs was 2.00 (95% CI [1.58–2.55]). The test of group differences was not significant (*Q* = 2.69, *p* = 0.26), indicating that study design did not significantly moderate the relationship between sport specialization and injury risk (see Fig. S1).

### The moderating effect of sports type

Athletes participating in contact sports demonstrated significantly greater odds of injury, with a pooled OR of 2.67 (95% CI [2.05–3.48], *p* < 0.001). For those involved in multiple sports, the pooled OR was 1.61 (95% CI [1.17–2.20], *p* < 0.001), while non-contact sports athletes showed a pooled OR of 2.41 (95% CI [1.78–3.26], *p* < 0.001). The overall pooled estimate across all sport types was 2.00 (95% CI [1.58–2.55], *p* < 0.001). A test of subgroup differences revealed statistically significant variation among the three sport type groups (*Q* = 6.17, *p* = 0.049), indicating that sport type significantly moderated the relationship between sport specialization and injury risk (see Fig. S2). *Post hoc* pairwise comparisons were conducted to further examine differences between sport type groups. The results indicated that athletes participating in contact sports had significantly higher odds of injury compared to those participating in multiple sports (*p* = 0.004). Similarly, athletes in non-contact sports also demonstrated significantly higher odds of injury than those in multiple sports (*p* = 0.037). However, no significant difference was observed between contact and non-contact sports (*p* = 0.55).

### The moderating effect of chronological age

Participants’ ages were categorized into two developmental groups: early adolescence (10–13 years) and middle-to-late adolescence (14–18 years). Based on the meta-analytic findings, middle-to-late adolescence was associated with a pooled injury risk of OR = 2.18 (95% CI [1.69–2.80], *p* < 0.001), whereas early adolescence yielded OR = 1.75 (95% CI [1.09–2.80], *p* = 0.01). The overall pooled estimate was OR = 2.00 (95% CI [1.58–2.55], *p* < 0.001). The test of subgroup differences was not statistically significant (*Q* = 0.64, *p* = 0.42), indicating that although middle-to-late adolescence showed a higher point estimate, the difference between age groups did not reach statistical significance (see Fig. S3).

### The moderating effect of sex

Results indicated that female athletes had an injury risk with an OR of 2.39 (95% CI [1.78–3.20], *p* < 0.001), while the male subgroup included only one study and yielded an of 3.77 (95% CI [1.39–10.20], *p* = not available, *n* = 1). The overall pooled OR was 2.48 (95% CI [1.87–3.28], *p* < 0.001), and the test of group difference was not significant (*Q* = 0.74, *p* = 0.39), indicating no statistically significant difference in injury risk between female and male athletes in this analysis (see Fig. S4).

### The moderating effect of injury measurement type

The moderating effect of injury measurement type was examined by categorizing studies into three subgroups: clinical diagnosis, mix, and self-report. Studies using clinical diagnosis yielded a pooled injury risk of OR = 2.06 (95% CI [1.29–3.29], *p* < 0.001), accompanied by moderate to high heterogeneity (*τ*^2^ = 0.10, *I*^2^ = 61.24%). Studies using mixed validated measurement (mix) demonstrated a pooled OR of 1.89 (95% CI [1.32–2.69], *p* < 0.001), with high heterogeneity (*τ*^2^ = 0.19, *I*^2^ = 75.91%). Studies relying on self-report showed a pooled OR of 2.58 (95% CI [1.66–4.00], *p* < 0.001), with no observed heterogeneity (*τ*^2^ <0.01, *I*^2^ <0.01%). The overall pooled estimate across all three measurement types was OR = 2.00 (95% CI [1.58–2.55], *p* < 0.001). The test of subgroup differences was not statistically significant (*Q* = 1.20, *p* = 0.55), indicating that the distinction between clinical diagnosis, mixed validated measurement, and self-report methods did not significantly influence the observed association between sport specialization and injury risk (see Fig. S5).

### The moderating effect of injury mechanism

The moderating effect of injury mechanism was examined by categorizing studies into two subgroups: overuse injuries and mixed injury outcomes. Overuse injuries referred to chronic overload-related, gradual-onset, or serious overuse injuries, whereas mixed injury outcomes referred to non-specific musculoskeletal injuries, concussions, stress fractures, or injury outcomes that could not be confidently classified as purely overuse-related based on the original article. Based on the meta-analytic results, mixed injury outcomes were associated with a pooled injury risk of OR = 2.48 (95% CI [2.01–3.05], *p* < 0.001), whereas overuse injuries yielded a pooled OR of 1.82 (95% CI [1.33–2.48], *p* < 0.001). The overall pooled estimate across all included studies was OR = 2.00 (95% CI [1.58–2.55], *p* < 0.001). The test of subgroup differences was not statistically significant (*Q* = 2.62, *p* = 0.11), indicating that while mixed injury outcomes exhibited a higher point estimate, the difference between injury mechanism subgroups did not reach statistical significance (see Fig. S6).

### The moderating effect of anatomical location of injury

Anatomical location of injury was categorized into two subgroups: lower extremity injuries and upper extremity injuries. Only studies in which injury location could be clearly classified as lower extremity or upper extremity were included in this subgroup analysis. Meta-analytic estimates revealed that lower extremity injuries were associated with a pooled OR of 2.61 (95% CI [1.94–3.53], *p* < 0.001), while upper extremity injuries yielded a pooled OR of 2.87 (95% CI [1.49–5.55], *p* = 0.002). Across all included studies, the overall pooled estimate was OR = 2.66 (95% CI [2.02–3.49], *p* < 0.001). The test for subgroup differences was not statistically significant (*Q* = 0.07, *p* = 0.80), indicating that although upper extremity injuries exhibited a slightly higher point estimate, the difference between anatomical location subgroups did not attain statistical significance (see Fig. S7).

## Discussion

The findings indicated that higher levels of specialization were consistently associated with an increased risk of injury. Importantly, the moderation analyses revealed that sport type significantly influenced the magnitude of this association, whereas no significant moderating effects were identified for study design, sex, age, injury measurement type, injury mechanism, or injury anatomical location. Because the included studies used heterogeneous injury definitions, the pooled estimate should be interpreted as reflecting overall sports injury risk rather than risk for any single injury subtype. The additional subgroup analyses by injury mechanism and anatomical location were therefore conducted to explore whether the broader pooled association differed across clinically relevant injury categories. These results carry several methodological and practical implications for researchers examining specialization-related injury risk.

### Implications

Adolescent participation in competitive sports continues to increase globally (Eime et al., 2016). Such involvement is typically associated with physical, psychological, and social development (Gill, Williams & Reifsteck, 2017). Although sport specialization is not inherently detrimental, accumulating evidence indicates that it is associated with a higher likelihood of adverse outcomes. The present findings are consistent with earlier meta-analytic work on the specialization–injury association (Bell et al., 2018) and extend this body of evidence. Several mechanisms may explain the link between sport specialization and injury risk. Excessive training volume and intensity, particularly when repetitive movements target the same anatomical regions, impose substantial physical strain and may hinder healthy musculoskeletal development in youth athletes (Jayanthi et al., 2020). Beyond training load, adolescent athletes are exposed to a range of psychosocial stressors, including performance pressures, coach–athlete interactions, academic expectations, and family-related demands (Lu et al., 2012). Competitive sport participation also entails frequent travel, exposure to team dynamics, and adaptation to competitive environments (Arnold, Fletcher & Daniels, 2013). Prior research suggests that specialized athletes may encounter greater psychosocial demands than their non-specialized counterparts (Brenner et al., 2019; Malina, 2010). Moreover, meta-analytic evidence demonstrates that heightened life stress increases the risk of sport injury (Chyi et al., 2024), further contributing to the vulnerability of specialized youth athletes.

Contrary to the original expectation that study design would be a statistically significant moderator, the present findings showed that the specialization–injury association remained relatively stable across case-control, cohort, and cross-sectional studies. Although the pooled OR was numerically highest in the cohort subgroup, followed by the cross-sectional and case-control subgroups, the between-group difference was not statistically significant. This pattern suggests that the positive association between sport specialization and injury risk is not restricted to one specific observational design, but can be observed across multiple research approaches. At the same time, these subgroup differences should still be interpreted cautiously because the cohort estimate was based on only one study, limiting the precision and generalizability of that comparison. This pattern aligns with methodological principles emphasizing that the strength of causal inference varies by study design and measurement processes (Hess, 2023; Olier et al., 2023). Prospective cohort studies track exposure prior to injury occurrence, establishing temporal ordering and minimizing recall or reverse-causation bias (Olier et al., 2023). Cross-sectional studies assess exposure and outcomes simultaneously, which increases the risk of misinterpretation due to altered specialization behaviors after injury (Hess, 2023). Case-control studies depend on retrospective reporting of past specialization, making them vulnerable to recall error and differential misclassification. Variability in injury surveillance systems and exposure measurement further increases heterogeneity across studies, as highlighted by recent methodological consensus statements advocating for standardized prospective monitoring (Nielsen et al., 2020). Accordingly, even though study design did not significantly moderate the pooled association in the present analysis, methodological rigor remains highly relevant for interpreting the evidence base. The most robust estimates are likely to arise from well-conducted longitudinal studies using consistent exposure and injury definitions, and additional research employing such designs would substantively strengthen the evidence base (Olier et al., 2023; Sprouse et al., 2024).

Sport type significantly moderated the specialization–injury association. Specifically, contact sports (OR = 2.67) and non-contact sports (OR = 2.41) both showed significantly higher injury risks than the multiple-sport category (OR = 1.61), whereas the difference between contact and non-contact sports was not statistically significant. This finding refines the interpretation of specialization-related injury risk. Rather than suggesting that one particular sport context is uniquely problematic, the results indicate that specialization within a single sport context, whether contact or non-contact, may be more strongly associated with injury risk compared to participation patterns spanning multiple sports. This pattern is theoretically consistent with the idea that participation in multiple sports may reflect exposure patterns associated with lower injury risk, including diversified movement experiences and reduced repetitive strain on the same anatomical regions. In contrast, athletes specializing within one sport may be repeatedly exposed to similar biomechanical demands, which may increase the likelihood of both overuse and broader musculoskeletal injuries. This interpretation is consistent with prior systematic review evidence suggesting that sport sampling or diversified participation may be associated with lower injury risk compared with early specialization (Carder et al., 2020).

At the same time, the absence of a significant difference between contact and non-contact sports is noteworthy. This pattern contrasts with prior evidence showing higher injury incidence in contact sports in general injury epidemiology research (Koh, Cassidy & Watkinson, 2003; Montalvo et al., 2019). One possible explanation is that the present meta-analysis examined the association between specialization and injury within sport-type subgroups, rather than comparing the absolute injury incidence of contact *versus* non-contact sports *per se*. Thus, the results do not indicate that contact and non-contact sports carry identical injury burdens overall; rather, they suggest that the relative increase in injury risk associated with specialization may be similarly elevated in both single-sport contexts.

Another important consideration is that the multiple-sport subgroup included studies in which participants were drawn from more than one sport type, including both contact and non-contact activities. Accordingly, this subgroup may reflect greater variability in exposure patterns, movement demands, and training structures. While this limits the precision of direct sport-type interpretation, it also reinforces the broader observation that diversified sport participation may be associated with a weaker specialization–injury relationship. Future research should more directly compare single-sport specialists and multisport participants within the same sport systems to clarify whether the observed protective pattern is attributable to diversification itself or to other contextual differences in training exposure.

Sex did not significantly moderate the association between sport specialization and injury, despite theoretical expectations that anatomical, hormonal, and biomechanical differences may contribute to sex-specific injury vulnerability (Hewett, 2000; Larwa et al., 2021). Although effect sizes varied slightly by sex, these differences were not statistically significant. This finding does not contradict broader literature showing higher rates of certain injuries in female athletes, such as anterior cruciate ligament injuries (Ireland, 2002). Rather, it suggests that within the context of sport specialization, shared mechanisms, including repetitive loading, reduced movement variability, and intensive training demands, may play a more dominant role in injury risk than sex-related biological factors. However, this result should be interpreted conservatively because the male subgroup was represented by only one study, which substantially limits statistical power and the stability of subgroup comparisons. Future research should incorporate larger, sex-balanced samples and examine whether sex interacts with additional moderators (*e.g.*, sport type or training volume) to shape specialization-related injury risk.

Age did not significantly moderate the specialization–injury association, although middle-to-late adolescence showed a numerically higher pooled OR compared with early adolescence. This pattern suggests that specialization-related injury risk may extend across adolescence due to shared developmental vulnerabilities, including incomplete musculoskeletal maturation, ongoing growth processes, and insufficient neuromuscular stability (DiFiori et al., 2014; Jayanthi et al., 2015). Because the present study used chronological age categories rather than direct indicators of biological maturation, these findings should not be interpreted as evidence about puberty stage *per se*. The commonly used broad age classifications (“early adolescence” *vs.* “middle-late adolescence”) may obscure more precise developmental differences relevant to injury susceptibility. Future research should employ biologically informed staging methods, such as Tanner maturation stages, to more accurately identify developmental periods during which sport specialization confers elevated injury risk (DiFiori et al., 2014).

Injury measurement type (clinical diagnosis, mix, and self-report) also did not significantly moderate the specialization–injury association. This stability is notable because clinical diagnoses and self-reported injuries differ in sensitivity, particularly for mild or subclinical injuries (Bell et al., 2016; McGuine et al., 2017). The mix measurement subgroup, which integrates both clinical and self-reported data, exhibited the highest level of heterogeneity (*I*^2^ = 75.91%), suggesting that variability in how combined measurement approaches were operationalized across studies may contribute to between-study inconsistency. The absence of significant moderation indicates that specialization-related injury risk is consistently observed regardless of injury ascertainment method, suggesting that the observed association was relatively consistent across injury ascertainment approaches. At the same time, this result should be interpreted cautiously due to the relatively small number of studies within each subgroup and the unequal distribution of measurement types across studies. The inclusion of a mix measurement category in the present study further reflects the methodological heterogeneity of injury assessment in the current literature, which may partially explain the overall between-study variability observed in the meta-analysis. Future research should adopt standardized injury surveillance frameworks and, where possible, integrate clinical verification with validated self-report measures to improve measurement precision and comparability across studies.

The additional subgroup analyses by injury mechanism and anatomical location provide a further examination of outcome heterogeneity. Injury mechanism did not significantly moderate the specialization–injury association, although mixed injury outcomes showed a numerically higher pooled estimate than overuse injuries. This finding suggests that the association between sport specialization and injury was not limited to outcomes explicitly classified as overuse injuries. However, mixed injury outcomes included clinically diverse categories, such as non-specific musculoskeletal injuries, concussions, stress fractures, and combined injury outcomes, and therefore this subgroup should not be interpreted as representing a single injury mechanism. Rather, it reflects outcomes that were either non-overuse injuries or could not be confidently classified as purely overuse-related based on the primary reports.

Similarly, anatomical location did not significantly moderate the specialization–injury association. Both lower extremity and upper extremity subgroups showed elevated odds, suggesting that specialization-related injury risk may not be restricted to one anatomical region. However, this analysis included only studies with clearly classifiable injury locations and should be interpreted cautiously due to the limited number of studies and the potential for sport-specific exposure patterns. For example, throwing sports may produce greater upper extremity loading, whereas running and field-based sports may place greater demands on the lower extremity. Therefore, these subgroup analyses should be viewed as exploratory tests of outcome heterogeneity rather than definitive evidence that sport specialization affects all injury mechanisms or anatomical locations equally.

Overall, the present findings indicate that sport specialization is associated with increased injury risk across youth athletes, and that this relationship appears to be particularly shaped by sport participation structure. More specifically, the results suggest that specialization within a single sport context, whether contact or non-contact, may be associated with greater injury risk than participation patterns involving multiple sports. In contrast, the non-significant moderating effects of study design, sex, age, injury measurement, injury mechanism, and injury anatomical location type suggest that the specialization–injury association is relatively robust across these study characteristics. However, given the clinical heterogeneity of injury outcomes and the limited number of studies in several subgroups, these moderator findings should be interpreted as exploratory.

### Strengths

Present study has several notable strengths. First, the literature screening process was grounded in the definition and criteria proposed by Jayanthi et al. (2015), while also accommodating studies that adapted or expanded these criteria for specific research contexts. This approach allowed for consistency in core inclusion principles while maintaining sufficient flexibility to capture relevant variations in operational definitions. Second, two validated quality appraisal tools were employed: the JBI Critical Appraisal Checklist for Analytical Cross-Sectional Studies for cross-sectional studies and the Newcastle–Ottawa Scale for cohort and case-control studies (Aromataris et al., 2024; Moola et al., 2024; Wells et al., 2000). The application of these tools ensured that only methodologically robust studies were incorporated into the meta-analysis. Third, unlike prior syntheses that focused primarily on overuse injury, the present study adopted a broader injury framework, thereby better reflecting the heterogeneity of injury definitions in the current literature. To further address this heterogeneity, additional subgroup analyses by injury mechanism and anatomical location were conducted to examine whether the overall association differed across clinically relevant injury categories. Fourth, in addition to updating the pooled estimate of the specialization–injury association identified in earlier work (Bell et al., 2018), this study evaluated multiple potential moderators, including sport type, age, sex, injury measurement type, study design, injury mechanism, and injury anatomical location, and further conducted *post hoc* comparisons for sport type. This approach provided a more nuanced assessment of heterogeneity within the current evidence base. Finally, potential overlap between study samples was explicitly examined and addressed during data extraction, which strengthened the independence of the pooled effect sizes and improved the internal consistency of the meta-analysis.

### Limitations

This study has several limitations that should be acknowledged. First, the literature search was restricted to peer-reviewed articles published in English, which may have introduced language bias by excluding potentially relevant studies published in other languages. Although this approach is common in systematic reviews, it may limit the comprehensiveness of the synthesized evidence. Second, study inclusion was confined to research that operationalized sport specialization based on the framework proposed by Jayanthi et al. (2015). While this decision enhanced conceptual consistency across included studies, it also resulted in the exclusion of investigations that employed alternative definitions or dichotomous classifications of specialization (*e.g.*, specialized *vs.* non-specialized). Consequently, the findings may not fully represent the broader spectrum of specialization conceptualizations used in the literature. Third, the present meta-analysis was based on a relatively limited number of independent effect sizes, which reduced statistical power for detecting moderation effects. This limitation is particularly relevant for subgroup analyses involving sport type and sex, where certain categories were underrepresented. For example, the male subgroup was represented by only one study, and the cohort subgroup also included only one study, which limits the precision of those comparisons. In addition, high-contact sports such as wrestling and rugby, which are associated with elevated injury risk, were sparsely represented in the included studies, thereby constraining the generalizability of the findings across diverse sporting contexts. Moreover, the “multiple sports” category, although useful for capturing diversified sport participation, incorporated studies sampling athletes from more than one sport type and therefore may have combined heterogeneous exposure contexts. This classification was analytically necessary, but it may also have reduced conceptual specificity when interpreting sport-type effects. Fourth, an important limitation concerns the aggregation of clinically heterogeneous injury outcomes. Although a broader sports injury framework was adopted to reflect the diversity of the existing literature and to estimate overall injury burden, the included outcomes varied substantially in mechanism, anatomical location, onset, severity, and diagnostic criteria. Therefore, the pooled effect estimate should not be interpreted as applying uniformly across specific injury types, such as overuse injuries, concussions, stress fractures, or region-specific injuries (*e.g.*, lower *vs.* upper extremity injuries). Although subgroup analyses based on injury mechanism and anatomical location were conducted to partially address this issue, these analyses were constrained by the limited number of studies in several categories and should be considered exploratory. Finally, the broad age categories adopted in many primary studies (*e.g.*, early adolescence *versus* middle to late adolescence) may have obscured developmentally specific variations in injury risk. Adolescence is characterized by substantial interindividual variability in biological maturation, and chronological age alone may be insufficient to capture these differences. Future research should incorporate more precise indicators of maturation, such as Tanner staging or growth-related biomarkers, to better identify developmental periods during which sport specialization confers heightened injury vulnerability (Malina, Bouchard & Bar-Or, 2004). In addition, the certainty of the evidence was not formally assessed using established frameworks. This decision was based on the observational nature of the included studies, heterogeneity in study designs, and variability in outcome measurement across studies. As a result, the pooled estimates should be interpreted with appropriate caution, and future research employing longitudinal designs and standardized injury surveillance methods is needed to strengthen confidence in the evidence base.

### Future research and suggestions

Only one included study employed a cohort design to examine the longitudinal development of the specialization–injury relationship (Buser et al., 2025), as potentially overlapping studies were excluded to preserve independence of effect sizes. Cohort designs provide clearer temporal ordering between exposure and outcome and therefore may strengthen interpretation of longitudinal associations. Additional longitudinal studies using this approach would substantially strengthen the evidence base. Beyond training-related factors, youth athletes are influenced by a range of psychosocial variables that may increase or decrease injury risk. Future research should evaluate factors such as life stress, coping resources, and personal strengths, including attributes such as mental toughness, to determine whether these characteristics modify the specialization–injury association. Moreover, the use of multiple sources of injury data, including medical records, athletic care documentation, self-reported information, and electronic medical records, could enhance the accuracy and completeness of injury surveillance. Integrating these data sources would provide a more comprehensive understanding of injury patterns in specialized youth athletes. Future studies should also more directly compare single-sport specialists and multisport participants within comparable sport systems, and should distinguish diversified participation from broad multi-sport sampling across dissimilar activity types. Such work would help clarify whether the lower injury risk observed in the multiple-sport subgroup reflects true protective effects of diversification, or broader contextual differences in training structure and exposure.

## Conclusions

Youth sport specialization remains an important concern within competitive athletic environments. The findings of this systematic review and meta-analysis indicate a positive association between higher levels of specialization and increased sports injury risk, with variation across sport participation contexts. More specifically, athletes specializing within contact and non-contact sport contexts demonstrated higher pooled odds of injury than those represented in the multiple-sport subgroup. However, given the observational design, heterogeneous injury outcomes, and limited subgroup data in several analyses, these findings should be interpreted cautiously and should not be considered evidence of causal relationships.

##  Supplemental Information

10.7717/peerj.21680/supp-1 Supplemental Information 1 PRISMA checklist

10.7717/peerj.21680/supp-2 Supplemental Information 2 PRISMA abstract checklist

10.7717/peerj.21680/supp-3 Supplemental Information 3 Detailed search strategy

10.7717/peerj.21680/supp-4 Supplemental Information 4 Audit table

10.7717/peerj.21680/supp-5 Table S1 Quality assessment of the 15 included studies

10.7717/peerj.21680/supp-6 Supplemental Information 6 Supplemental figures of the moderation analysis

10.7717/peerj.21680/supp-7 Supplemental Information 7 Intended audience

10.7717/peerj.21680/supp-8 Data S1 Raw data

## Additional Information and Declarations

### Data Availability

The following information was supplied regarding data availability: This is a Systematic review/Meta-analysis.
