# Supplementary material for: Association between youth athletes’ sports specialization and injuries: a systematic review and meta-analysis
Source: PeerJ. 2026 Sep 7;14:e21680. doi: 10.7717/peerj.21680 (PMC13564398; doi:10.7717/peerj.21680)
Supplement: Supplementary file 3 [file peerj-14-21680-s003.docx]

Supplementary: Detailed search strategy

**1. Overview**

A comprehensive literature search was conducted to identify studies examining the association between sport specialization and injury in youth athletes. The search strategy was developed based on three key concepts:

Population: youth athletes.

Exposure: sport specialization.

Outcome: sports injury.

Boolean operators (AND, OR), phrase searching, and truncation (*) were applied where appropriate. The search strategy was adapted for each database according to its indexing system and search functions.

**2. Core Search String (General Strategy)**

(adolescent OR youth OR teenager OR "young athlete*" OR "adolescent athlete*" OR "youth sport*")

AND

("sport specialization" OR "sports specialization" OR "athletic specialization" OR specialization OR "specialized training" OR "single sport" OR "early specialization")

AND

("sports injury" OR "sport injury" OR "athletic injury" OR injury OR injuries OR trauma OR "overuse injury" OR "musculoskeletal injury")

**3. Database-Specific Search Strings**

(1) PubMed

((adolescent[Title/Abstract] OR youth[Title/Abstract] OR teenager[Title/Abstract] OR "young athlete*"[Title/Abstract] OR "adolescent athlete*"[Title/Abstract] OR "youth sport*"[Title/Abstract]))

AND

(("sport specialization"[Title/Abstract] OR "sports specialization"[Title/Abstract] OR "athletic specialization"[Title/Abstract] OR specialization[Title/Abstract] OR "specialized training"[Title/Abstract] OR "single sport"[Title/Abstract] OR "early specialization"[Title/Abstract]))

AND

(("sports injury"[Title/Abstract] OR "sport injury"[Title/Abstract] OR "athletic injury"[Title/Abstract] OR injury[Title/Abstract] OR injuries[Title/Abstract] OR trauma[Title/Abstract] OR "overuse injury"[Title/Abstract] OR "musculoskeletal injury"[Title/Abstract]))

(2) Web of Science

TS = (

(adolescent OR youth OR teenager OR "young athlete*" OR "adolescent athlete*" OR "youth sport*")

AND

("sport specialization" OR "sports specialization" OR "athletic specialization" OR specialization OR "specialized training" OR "single sport" OR "early specialization")

AND

("sports injury" OR "sport injury" OR "athletic injury" OR injury OR injuries OR trauma OR "overuse injury" OR "musculoskeletal injury")

)

(3) Scopus

TITLE-ABS-KEY (

(adolescent OR youth OR teenager OR "young athlete*" OR "adolescent athlete*" OR "youth sport*")

AND

("sport specialization" OR "sports specialization" OR "athletic specialization" OR specialization OR "specialized training" OR "single sport" OR "early specialization")

AND

("sports injury" OR "sport injury" OR "athletic injury" OR injury OR injuries OR trauma OR "overuse injury" OR "musculoskeletal injury")

)

(4) ScienceDirect / Sage / Taylor & Francis / Ovid

(adolescent OR youth OR teenager OR "young athlete*" OR "adolescent athlete*" OR "youth sport*")

AND

("sport specialization" OR "sports specialization" OR "athletic specialization" OR specialization OR "specialized training" OR "single sport" OR "early specialization")

AND

("sports injury" OR "sport injury" OR "athletic injury" OR injury OR injuries OR trauma OR "overuse injury" OR "musculoskeletal injury")

(5) Google Scholar

(adolescent OR youth athlete) AND ("sport specialization") AND (injury)

Results were screened based on relevance, and the first several hundred records were examined.

**4. Search Limits**

Language: English.

Publication type: Peer-reviewed journal articles.

Time frame: From database inception to March 20, 2026.

**5. Additional Search Procedures**

Reference lists of included studies and relevant review articles were manually screened to identify additional eligible studies.

Duplicate records were removed prior to screening.
