# Supplementary material for: Association between youth athletes’ sports specialization and injuries: a systematic review and meta-analysis
Source: PeerJ. 2026 Sep 7;14:e21680. doi: 10.7717/peerj.21680 (PMC13564398; doi:10.7717/peerj.21680)
Supplement: Supplementary file 4 [file peerj-14-21680-s004.docx]

Supplementary: Audit table

Audit table of effect sizes, coding decisions, and moderator inclusion

This table provides a comprehensive audit of all included studies, including extracted effect sizes, coding decisions for moderators, and their inclusion across subgroup analyses. All effect sizes reported in the manuscript, tables, and figures were generated from a single finalized dataset to ensure full numerical consistency and reproducibility.

| Study | Design | Sport Type | Age Group | Sex | Injury Type | Measure | Injury Mechan-ism | Injury Location | OR | 95% CI | Moderation Test Included | | | | | | |
| --- | --- | --- | --- | --- | --- | --- | --- | --- | --- | --- | --- | --- | --- | --- | --- | --- | --- |
|  |  |  |  |  |  |  |  |  |  |  | Study Design | Sport Type | Age | Sex | Meas-ure | Injury Mechanism | Injury Location |
| Bell et al. (2016) | Cross-sectional | Contact | Middle-Late | Mixed | LE musculoskelet-al | Mix | Overuse Injuries | Lower extremity | 2.93 | 1.16–7.36 | ✔ | ✔ | ✔ | ✖ | ✔ | ✔ | ✔ |
| Biese et al. (2020a) | Cross-sectional | Non-contact | Middle-Late | Female | Musculoskelet-al | Mix | Mix | NR | 2.30 | 1.64–3.23 | ✔ | ✔ | ✔ | ✔ | ✔ | ✔ | ✖ |
| Biese et al. (2020b) | Cross-sectional | Multiple | Early | Mixed | Acute + Overuse | Mix | Overuse Injuries | NR | 1.50 | 1.11–2.02 | ✔ | ✔ | ✔ | ✖ | ✔ | ✔ | ✖ |
| Buser et al. (2025) | Cohort | Multiple | Middle-Late | Mixed | Overuse | Clinical | Overuse Injuries | NR | 3.19 | 1.69–6.03 | ✔ | ✔ | ✔ | ✖ | ✔ | ✔ | ✖ |
| Jayanthi et al. (2015) | Case-control | Multiple | Early | Mixed | Overuse | Clinical | Overuse Injuries | NR | 2.25 | 1.27–3.99 | ✔ | ✔ | ✔ | ✖ | ✔ | ✔ | ✖ |
| Jayanthi et al. (2020) | Case-control | Multiple | Middle-Late | Mixed | All + Overuse | Clinical | Overuse Injuries | NR | 1.46 | 1.04–2.04 | ✔ | ✔ | ✔ | ✖ | ✔ | ✔ | ✖ |
| Lear et al. (2024) | Cross-sectional | Non-contact | Middle-Late | Female | UE overuse | Self-report | Overuse Injuries | Upper extremity | 2.33 | 0.97–5.60 | ✔ | ✔ | ✔ | ✔ | ✔ | ✔ | ✔ |
| McGowan et al. (2019) | Cross-sectional | Multiple | Early | Mixed | Gradual onset | Mix | Overuse Injuries | NR | 0.75 | 0.49–1.15 | ✔ | ✔ | ✔ | ✖ | ✔ | ✔ | ✖ |
| Okoruwa et al. (2022) | Cross-sectional | Multiple | Middle-Late | Female | Stress fracture + concussion | Self-report | Mix | NR | 2.93 | 1.38–6.23 | ✔ | ✔ | ✔ | ✔ | ✔ | ✔ | ✖ |
| Post et al. (2017) | Cross-sectional | Contact | Middle-Late | Mixed | LE injury | Mix | Mix | Lower extremity | 2.58 | 1.88–3.54 | ✔ | ✔ | ✔ | ✖ | ✔ | ✔ | ✔ |
| Post et al. (2020a) | Cross-sectional | Multiple | Middle-Late | Mixed | Overuse | Mix | Overuse Injuries | NR | 1.20 | 0.75–1.91 | ✔ | ✔ | ✔ | ✖ | ✔ | ✔ | ✖ |
| Post et al. (2020b) | Cross-sectional | Non-contact | Middle-Late | Male | UE overuse | Mix | Overuse Injuries | Upper extremity | 3.77 | 1.39–10.20 | ✔ | ✔ | ✔ | ✔ | ✔ | ✔ | ✔ |
| Post et al. (2021) | Cross-sectional | Contact | Early | Mixed | Musculoskelet-al | Self-report | Mix | NR | 2.47 | 1.25–4.88 | ✔ | ✔ | ✔ | ✖ | ✔ | ✔ | ✖ |
| Whatman et al. (2021) | Cross-sectional | Multiple | Early | Mixed | Musculoskelet-al | Mix | Mix | NR | 2.21 | 0.43–11.36 | ✔ | ✔ | ✔ | ✖ | ✔ | ✔ | ✖ |
| Zoellner et al. (2022) | Cross-sectional | Contact | Early | Mixed | Gradual -onset | Mix | Overuse Injuries | NR | 4.17 | 1.48–11.74 | ✔ | ✔ | ✔ | ✖ | ✔ | ✔ | ✖ |

*Note*. ‘✔’ indicates that the study was included in the corresponding moderator analysis. Studies with mixed-sex samples were not included in sex-based subgroup analyses. Sport type classified as ‘Multiple’ refers to participation in more than one sport, which may include both contact and non-contact sports. Injury mechanism classified as ‘overuse injury’ refers to caused by repetitive microtrauma without an acute traumatic event, while ‘Mix’ refers to non-specific musculoskeletal injuries, concussions, and stress fractures. ‘NR’ in injury location refers to ‘not recorded’ in study.
