# Supplementary material for: Association between youth athletes’ sports specialization and injuries: a systematic review and meta-analysis
Source: PeerJ. 2026 Sep 7;14:e21680. doi: 10.7717/peerj.21680 (PMC13564398; doi:10.7717/peerj.21680)
Supplement: Supplementary file 5 [file peerj-14-21680-s005.docx]

Supplementary: Table S1

*Quality assessment of the 15 included studies*

| Author (year) | Study Design | A | B | C | D | E | F | G | H | JBI/NOS Score | Quality Assessment |
| --- | --- | --- | --- | --- | --- | --- | --- | --- | --- | --- | --- |
| Bell et al. (2016) | Cross-sectional | √ | √ | O | √ | √ | √ | O | √ | 6 | High |
| Biese et al. (2020a) | Cross-sectional | √ | √ | √ | √ | √ | √ | O | √ | 7 | High |
| Biese et al. (2020b) | Cross-sectional | √ | √ | O | √ | √ | √ | O | √ | 6 | High |
| Buser et al. (2025) | Cohort | √ | √ | √ | × | √ | √ | √ | × | 6 | High |
| Jayanthi et al. (2015) | Case-control | √ | × | √ | √ | √√ | × | √ | × | 6 | High |
| Jayanthi et al. (2020) | Case-control | √ | × | √ | √ | √√ | × | √ | × | 6 | High |
| Lear et al. (2024) | Cross-sectional | √ | √ | O | √ | √ | √ | O | √ | 6 | High |
| McGowan et al. (2019) | Cross-sectional | √ | √ | O | √ | √ | √ | O | √ | 6 | High |
| Okoruwa et al. (2022) | Cross-sectional | √ | √ | O | √ | √ | √ | O | √ | 6 | High |
| Post et al. (2017) | Cross-sectional | √ | √ | O | √ | √ | √ | O | √ | 6 | High |
| Post et al. (2020a) | Cross-sectional | √ | √ | O | √ | √ | √ | O | √ | 6 | High |
| Post et al. (2020b) | Cross-sectional | √ | √ | √ | √ | √ | √ | O | √ | 7 | High |
| Post et al. (2021) | Cross-sectional | √ | √ | O | √ | √ | √ | O | √ | 6 | High |
| Whatman et al. (2021) | Cross-sectional | √ | √ | O | √ | √ | √ | O | √ | 6 | High |
| Zoellner et al. (2022) | Cross-sectional | √ | √ | O | √ | √ | √ | O | √ | 6 | High |

*Note.* For cross-sectional studies, quality assessment was conducted using the JBI Critical Appraisal Checklist for Analytical Cross-Sectional Studies. √ = Yes; O = Unclear; × = No. Studies with more than five “yes” ratings were classified as high quality, those with three to five “yes” ratings as moderate quality, and those with fewer than three “yes” ratings as low quality. For cohort and case-control studies, methodological quality was evaluated using the Newcastle–Ottawa Scale (NOS), in which higher scores indicate better methodological quality.

Assessment Criteria: (1) Cross-sectional Studies: A: Were the criteria for inclusion in the sample clearly defined? B: Were the study subjects and the setting described in detail? C: Was the exposure measured in a valid and reliable way? D: Were objective, standard criteria used for measurement of the condition? E: Were confounding factors identified? F: Were strategies to deal with confounding factors stated? G: Were the outcomes measured in a valid and reliable way? H: Was appropriate statistical analysis used? (2) Cohort Studies: A: Representativeness of the exposed cohort? B: Selection of the non-exposed cohort? C: Ascertainment of exposure? D: Demonstration that outcome of interest was not present at start of study? E: Comparability of cohorts on the basis of the design or analysis? F: Assessment of outcome? G: Was follow-up long enough for outcomes to occur? H: Adequacy of follow up of cohort? (3) Case-control Studies: A: Is the case definition adequate? B: Representativeness of the cases? C: Selection of Controls? D: Definition of Controls? E: Comparability of cases and controls on the basis of the design or analysis? F: Ascertainment of exposure? G: Same method of ascertainment for cases and controls? H: Non-Response rate?
