# Supplementary material for: Association between youth athletes’ sports specialization and injuries: a systematic review and meta-analysis
Source: PeerJ. 2026 Sep 7;14:e21680. doi: 10.7717/peerj.21680 (PMC13564398; doi:10.7717/peerj.21680)
Supplement: Supplementary file 6 [file peerj-14-21680-s006.docx]

Supplementary: Figures

**Figure S1**

*Moderation analysis of specialization-injury grouped by study design*

**Figure S2**

*Moderation analysis of specialization-injury grouped by sports type*

**Figure S3**

*Moderation analysis of specialization-injury relationship grouped by adolescent age stage*

**Figure S4**

*Moderation analysis of specialization-injury grouped by sex*

**Figure S5**

*Moderation analysis of specialization-injury grouped by injury measurement type*

**Figure S6**

*Moderation analysis of specialization-injury grouped by injury mechanism*

**Figure S7**

*Moderation analysis of specialization-injury grouped by anatomical location*
