# Supplementary material for: Association between youth athletes’ sports specialization and injuries: a systematic review and meta-analysis
Source: PeerJ. 2026 Sep 7;14:e21680. doi: 10.7717/peerj.21680 (PMC13564398; doi:10.7717/peerj.21680)
Supplement: Supplementary file 7 [file peerj-14-21680-s007.docx]

Intended Audience for This Systematic Review and Meta-Analysis

This systematic review and meta-analysis is primarily intended for researchers, clinicians, and practitioners working in the fields of sports medicine, sport and exercise psychology, athletic training, and youth sport development. By synthesizing evidence on the association between sport specialization and overuse injuries in youth athletes, the study provides an updated and methodologically rigorous summary of injury risk patterns that are directly relevant to injury prevention and athlete health.

In addition, this review is intended to inform coaches, sport administrators, and policy makers involved in youth sport programming. The findings offer evidence-based insights that may support the development of training guidelines, participation policies, and early specialization recommendations aimed at reducing injury risk while promoting long-term athlete development. Researchers conducting future longitudinal or intervention-based studies may also benefit from the identified research gaps and methodological considerations highlighted in this review.
